# Exhaled breath condensate in intubated neonates- a window into the lung’s glutathione status

**DOI:** 10.1186/1465-9921-15-1

**Published:** 2014-01-07

**Authors:** Maria I Rosso, Susan Roark, Esther Taylor, XiaoDu Ping, Janine M Ward, Katherine Roche, Courtney McCracken, Lou Ann S Brown, Theresa W Gauthier

**Affiliations:** 1 Department of Pediatrics, Emory University School of Medicine, Atlanta, GA, USA; 2 Children’s Healthcare of Atlanta, Atlanta, GA, USA; 3 Children’s Center for Developmental Lung Biology, Children’s Healthcare of Atlanta, Atlanta, GA, USA

**Keywords:** Tracheal aspirate, Exhaled breath condensate, Prematurity, Glutathione

## Abstract

**Background:**

Analysis of exhaled breath condensates (EBC) is a non-invasive technique to evaluate biomarkers such as antioxidants in the pediatric population, but limited data exists of its use in intubated patients, particularly newborns. Currently, tracheal aspirate (TA) serves as the gold standard collection modality in critically ill newborns, but this method remains invasive. We tested the *hypothesis* that glutathione status would positively correlate between EBC and TA collections in intubated newborns in the Newborn Intensive Care Unit (NICU). We also hypothesized that these measurements would be associated with alveolar macrophage (AM) glutathione status in the newborn lung.

**Methods:**

Reduced glutathione (rGSH), glutathione disulfide (GSSG), and total GSH (rGSH + (2 X GSSG)) were measured in sequential EBC and TA samples from 26 intubated newborns via high performance liquid chromatography (HPLC). Additionally, AM glutathione was evaluated via immunofluorescence. Pearson’s correlation coefficient and associated 95% confidence intervals were used to quantify the associations between raw and urea-corrected concentrations in EBC and TA samples and AM staining. Statistical significance was defined as p ≤ 0.05 using two-tailed tests. The sample size was projected to allow for a correlation coefficient of 0.5, with 0.8 power and alpha of 0.05.

**Results:**

EBC was obtainable from intubated newborns without adverse clinical events. EBC samples demonstrated moderate to strong positive correlations with TA samples in terms of rGSH, GSSG and total GSH. Positive correlations between the two sampling sites were observed in both raw and urea-corrected concentrations of rGSH, GSSG and total GSH. AM glutathione staining moderately correlated with GSSG and total GSH status in both the TA and EBC.

**Conclusions:**

GSH status in EBC samples of intubated newborns significantly correlated with the GSH status of the TA sample and was reflective of cellular GSH status in this cohort of neonatal patients. Non-invasive EBC sampling of intubated newborns holds promise for monitoring antioxidant status such as GSH in the premature lung. Further studies are necessary to evaluate the potential relationships between EBC biomarkers in the intubated premature newborn and respiratory morbidities.

## Background

Bronchopulmonary dysplasia (BPD) is a common complication of survivors of extreme prematurity, causing significant morbidity and mortality among critically ill NICU patients [1,2]. The precise pathophysiology of lung injury remains under investigation but it remains clear that inflammation and oxidant stress amplify injury to the premature lung [3-5]. Antioxidant defenses such as glutathione (GSH) are decreased in the premature newborn lung, placing them at increased risk for cellular injury from increased production of reactive oxygen species [6]. Low levels of GSH in the plasma and the bronchoalveolar fluid (BAL) of premature newborns have been implicated in the development of BPD [7,8], and the availability of GSH modulates critical cellular functioning in the lung [9].

Current measurements of oxidant stress and inflammation include evaluation of samples obtained from airway lavage, with tracheal aspirate (TA) sampling being the primary site for the premature newborn [10,11]. However, this standard collection modality remains invasive for routine use in newborns, particularly those born prematurely. In recent years, analysis of the volatile and non-volatile constituents in exhaled breath condensate (EBC) has evolved as a novel means to non-invasively study oxidant markers and inflammatory mediators in the airway surface liquid [12-17]. Our laboratory has successfully used this method in previous studies to investigate oxidant stress markers in both asthmatic children [18] and adults [19].

Although TA samples are the mainstay of evaluating samples obtained from premature newborns, there is a lack of data regarding the correlation of the antioxidant status between the TA sample and a non-invasive EBC sample in neonates. We *hypothesized* that measurements of GSH status obtained via TA collection and EBC collection would positively correlate. Furthermore, since we and others have demonstrated that AM GSH status is dependent on the external milieu of GSH [9,20], we also hypothesized that the GSH status of the EBC and TA would be reflective of cellular GSH in isolated AM. Therefore the *goal* of this study was to compare the GSH status of a non-invasive EBC sample to its corresponding TA and AM sample to determine whether EBC was reflective of GSH within the neonatal lung and AM.

## Methods

### Subjects

Intubated newborns were enrolled from the NICU at Children’s Healthcare of Atlanta at Egleston (CHOA), a level IV intensive care unit within the Emory Division of Neonatology System, in the city of Atlanta from January 2011 through October 2012. Informed consent was obtained from guardians using procedures approved by the Institutional Review Board at Emory University School of Medicine (Gauthier, IRB00038093). All intubated babies were eligible for enrollment. Exclusion criteria included patients deemed too unstable by the attending neonatologist or those on high frequency ventilation, inhaled nitric oxide, or extracorporeal membrane oxygenation (ECMO). Clinical information was ascertained from the electronic medical record.

### Exhaled breath condensate (EBC) collection

**Figure 1:**
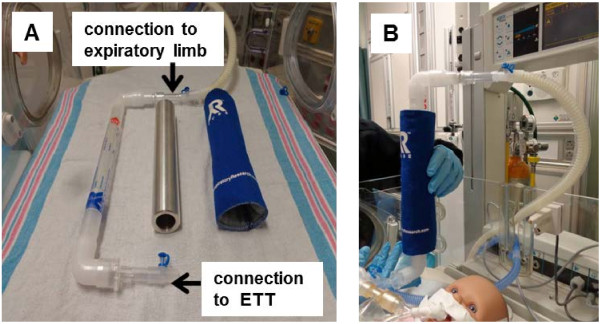
**EBC collection from the expiratory limb of the ventilator. A)** The RTUBE™ and its cooling sleeve covered by the blue insulated cover where connected to the expiratory limb of the ventilator circuit via valve-less connectors. **B)** A simulated collection of EBC on an intubated neonatal mannequin.

Liquid condensate was collected from the exhaled breath of mechanically ventilated newborns for 30 minutes. The EBC collection device consisted of an RTUBE™ (Respiratory Research, Inc. Austin, TX) encased in an aluminum cooling sleeve that was cooled to -80°C for at least 2 hours prior to use. A modified collection device was used to collect EBC off the ventilator circuit. The RTUBE™ and cooling sleeve, covered by a blue insulating cover, were connected to the ETT and to the expiratory limb of the ventilator circuit via valve-less connectors provided in most standard ventilator circuits (Figure 1A). Figure 1B depicts a simulated collection from an intubated neonatal mannequin. Immediately after collection, the condensate tubes were capped, placed on wet ice and transported to the laboratory for analyses. The process of EBC collection was monitored at the bedside by the investigators (MR, SR, ET) and was tolerated well by all subjects, without significant changes in heart rate, oxygen saturation or blood pressure.

### Tracheal aspirate (TA) collection

TA samples where obtained within one hour of EBC collection. For the suctioning procedure, bacteriostatic saline (~1 ml) was instilled into the trachea. After several ventilator breaths, an in-line suction catheter (Ballard, Kimberly-Clark, Irving, TX) was inserted just past the endotracheal tube and the sample was then retrieved into a closed, sterile Lukens trap (Covidien, Mansfield, MA). Additional bacteriostatic saline was used if necessary to clear the sample from the suction catheter. Samples were immediately placed on wet ice and transported to the laboratory for analyses.

### Sample processing and high performance liquid chromatography (HPLC) analysis

Patients and samples were de-identified with a study number to ensure confidentiality. All samples were transported to the laboratory on wet ice within an hour of collection. In previous studies, we determined that the rate of oxidation was ~5% per hour if the sample was maintained in a sterile Lukens trap on wet ice [21]. TA samples were centrifuged for 10 minutes to remove the cell pellet. EBC was extracted from the RTUBE with a plunger apparatus. To prevent auto-oxidation before analysis, 240 μL of the EBC was aliquoted into pre-made tubes containing 21 μL of a diluted preservative with a final concentration of 5% perchloric acid, 0.1 M boric acid, 6.7 mM iodoacetic acid (IAA), and 5 nM of the internal standard γ-glutamyl-glutamate as we have previously described [22]. For TA, 250 μL of sample was added to pre-made tubes containing 250 μL of preservative for a final concentration of 10% perchloric acid, 0.2 M boric acid, 13.4 mM IAA, and 10 μMγ-glutamyl-glutamate. All samples were stored at -80°C until batch analysis by HPLC, also previously described by this laboratory [21,23]. GSH and GSSG fractions were treated with dansyl chloride and the dansylated derivatives separated using an amino μBondaPak column (Waters, Milford MA) with fluorescent detection. The lower limits of detection were 15 fmol/μl for rGSH and were consistent with studies by Komatsu and Obata [24] which used similar methods and reported similar lower limits of detection. All of the samples in this study were above the noted limits of detection. To minimize operational variability, all samples from this study were analyzed within the same HPLC run. Inter-assay coefficient of variability was determined by performing HPLC analysis on a mixture of standards including cysteine, cystine, rGSH, and GSSG. This mixture was analyzed immediately before, in the middle and at the end of sample set analysis. Based on this cocktail of standards, the inter-assay coefficient of variability was calculated at 1%. Given the limited sample volume obtained, we were unable to calculate intra-assay variability.

The subject’s blood urea nitrogen level (BUN, mg/dl) was noted in the medical record on the day samples were obtained. Sample urea was measured using the Bethleot/ Colorimetric assay (Pointe Scientific, Canton, MI - sensitivity range of 0.05 to 150 mg/dl). Sample concentrations of rGSH, GSSG and total GSH (defined as: GSH + (2 X GSSG)) are presented as both raw values and urea corrected values. Samples were corrected for dilution by multiplication with the urea dilution factor (f) defined as f = Patient BUN/ sample urea used was as previously described for EBC samples [21].

### AM immunofluorescence

TA samples where centrifuged as noted above to obtain cells (300 g, 10 minutes). The supernatant was processed as above and the cell pellets where re-suspended in RPMI 1640 media with 10% fetal calf serum (FCS) and plated under sterile conditions for 1 hour. The media was removed and the adhered cells washed three times with phosphate buffered saline (PBS). Cells where then fixed with 4% paraformaldehyde solution for 30 minutes, washed with PBS and stored at 4°C. At the time of analysis non-specific binding was blocked with 10% bovine serum albumin (BSA, 1 hour) and the cells were stained for protein-glutathione adducts (primary mouse monoclonal IgG GSH, 2 hours) (Virogen, Watertown, MA). After washing, a secondary Alexa fluorescence 488 green anti-mouse IgG was added for 2 hours (Life Technologies, Carlsbad, CA). The cells were identified as AM by cell morphology and then analyzed for fluorescence via confocal fluorescent microscopy (Olympus FluoView FV1000). The cellular fluorescence was quantified as relative fluorescent units (RFU) /cell as we previously described [25].

### Statistical analyses

All data were analyzed with SPSS® for Windows software (Version 18.0, SPSS Inc., Chicago, IL) and SAS 9.3 (Cary, NC). Prior to analysis, data were assessed for normality using histograms, normal probability plots, and the Kolmogorov-Smirnov test of normality. For concentrations that were non-normally distributed, a log_10_ normalizing transformation was applied. Pearson’s correlation coefficient and associated 95% confidence intervals were used to quantify the association between TA and EBC concentrations. Scatter plots were used to assess whether the relationship between two concentrations appeared linear and to confirm that the Pearson’s correlation coefficient was an appropriate measure of association. Statistical significance was defined as p ≤ 0.05 using two-tailed tests. The sample size was determined in order to detect a minimum correlation coefficient of 0.5, with 0.8 power and an alpha of 0.05.

## Results

### Study population

Twenty-six (26) intubated newborns on mechanical ventilation were enrolled (Table 1). Enrolled subjects had a median birthweight of 1564 grams (interquartile range (IQR) 698, 2914 grams) and a median gestational age of 32.5 weeks (IQR: 24, 38 weeks). At the time of EBC and TA collection, the median weight was 2280 grams (IQR: 1500, 3254 grams) with a corrected gestational age of 35 weeks (IQR: 34, 39.5 weeks). Subjects were ventilated for a mean of 21 days (IQR: 7, 59.5 days) at the time of sample collection. As noted in Table 1, the majority of patients were admitted for surgical procedures.

**Table 1:** Patient demographics

**Subject number**	**GA (weeks)**	**GA at sample collection (weeks)**	**Birth weight (grams)**	**Weight at sample collection (grams)**	**BUN at sample collection (mg/dl)**	**Duration of mechanical ventilation (days)**	**Primary diagnosis**
1	37	40	2442	2760	3	21	Hypoglossia-hypodactylia syndrome
2	24	27	675	1000	6	21	Spontaneous intestinal perforation
3	26	35	686	1730	3	189	Patent ductus arteriosus
4	24	35	690	3780	8	77	Subglottic stenosis
5	35	42	1980	3000	12	49	Congenital diaphragmatic hernia
6	41	42	3530	4060	10	7	Esophageal atresia/tracheal esophageal fistula
7	39	40	3800	3850	5	7	Neonatal seizures
8	37	40	2782	3365	7	21	Laryngeal cleft
9	26	34	803	1650	5	56	Jejunal stricture
10	24	34	720	1895	1	70	Pneumoperitoneum
11	24	30	760	1380	18	42	Necrotizing enterocolitis
12	38	40	3100	2940	19	14	Gastroschisis
13	38	39	2858	2858	13	7	Gastroschisis
14	39	40	3080	3265	18	7	Imperforate anus/congenital heart disease
15	35	36	2440	2440	16	7	Gastroschisis
16	24	26	700	810	29	14	Spontaneous intestinal perforation
17	38	38	3590	3590	12	5	Congenital diaphragmatic hernia
18	31	33	1417	1100	26	14	Congenital heart disease, duodenal atresia
19	34	34	2120	2120	15	4	Multiple jejunal atresias
20	35	43	1710	2500	16	56	Intestinal perforation, microcolon
21	39	41	3170	3250	41	14	Pentalogy of cantrell
22	25	37	450	1530	33	84	Extreme prematurity
23	24	32	550	1200	18	56	Extreme prematurity
24	23	34	610	1410	2	77	Spontaneous intestinal perforation
25	27	35	1110	1900	3	56	Patent ductus arteriosus
26	25	35	850	1650	33	70	Jejunal perforation

### GSH status was measureable in both TA and EBC samples

The median volume recovered from the TA was 0.75 ml (IQR: 0.6, 1.75 ml) while the median EBC volume recovered after 30 minutes of sampling was 2.2 ml (IQR: 1.5, 2.5 ml). GSH, GSSG and total GSH were detected by HPLC in all samples (100%) in the nM range. The urea dilution factors, raw concentrations, and urea-corrected concentrations are presented in Table 2.

**Table 2:** rGSH, GSSG and total GSH are measurable in both TA and EBC samples

	**TA**	**EBC**
**Collected fluid volume (ml)**	0.75 (0.6, 1.75)	2.2 (1.5, 2.5)
**Sample urea (mg/dl)**	11.2 (3.8, 28.0)	3.3 (3.1, 28.3)
**Urea dilution factor**	0.69 (0.42, 3.77)	0.90 (0.49, 4.87)
**Sample GSH (raw values, nM)**		
**rGSH**	155 (71, 1087)	0.5 (0.4, 37)
**GSSG**	112.0 (7.9, 345)	1.0 (0.8, 10.9)
**Total GSH**	273 (107, 1831)	2.3 (2.1, 63.7)
**Sample GSH (urea-corrected values, nM)**		
**rGSH**	225 (107, 562)	3.8 (0.5, 12.2)
**GSSG**	45.5 (20.9, 191)	4.3 (1.3, 8.6)
**Total GSH**	289 (195, 884)	12 (3.6, 28.1)
Values are presented as median (IQR)		

### TA and EBC concentrations of rGSH and GSSG significantly correlated

**Figure 2:**
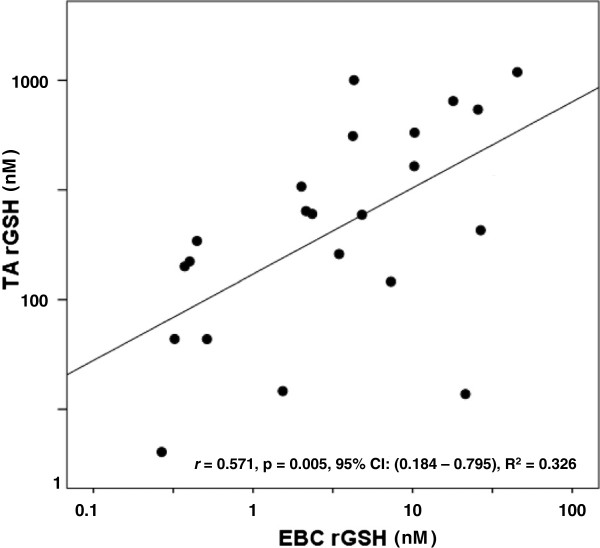
**Relationship between TA rGSH and EBC rGSH.** TA and EBC rGSH demonstrated a moderate positive correlation. Points represent the values of urea-corrected concentrations of rGSH in nM. Correlations were calculated after log_10_ normalization.

**Figure 3:**
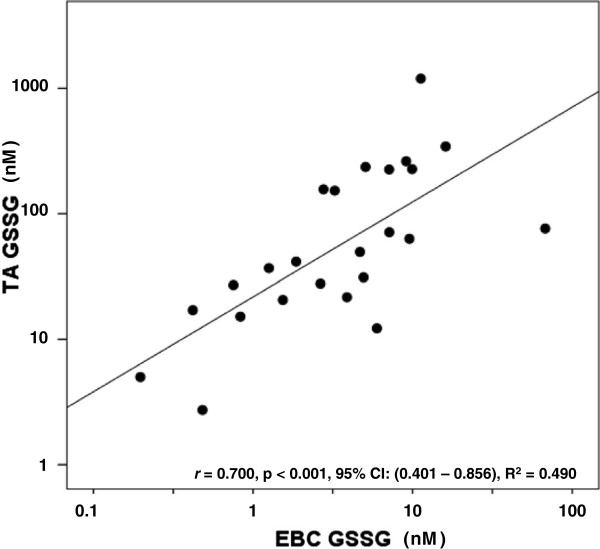
**Relationship between TA GSSG and EBC GSSG.** A strong positive correlation is seen between TA and EBC GSSG. Points represent the values of urea-corrected concentrations of GSSG in nM. Correlations were calculated after log_10_ normalization.

**Figure 4:**
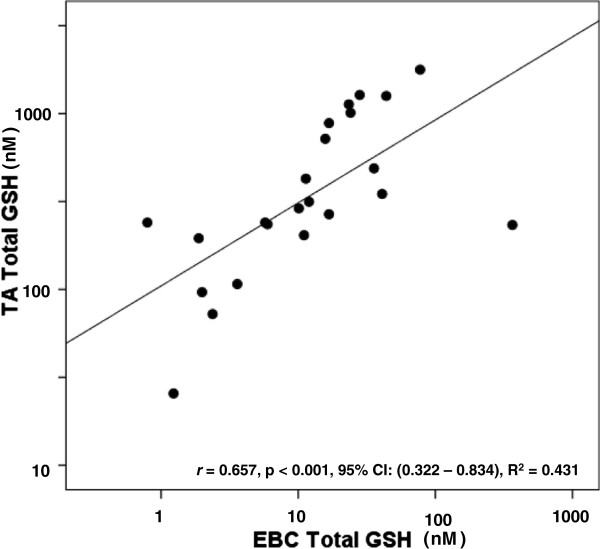
**Relationship between TA Total GSH and EBC Total GSH.** Total GSH (GSH + (2 X GSSG)) had a moderate positive correlation between the two sampling sites. Points represent the values of urea-corrected concentrations of total GSH in nM. Correlations were calculated after log_10_ normalization.

We postulated that rGSH and GSSG values measured in the TA would correlate with those measured via a non-invasive EBC. As seen in Figure 2, urea-corrected rGSH in the TA demonstrated a moderate positive correlation with urea-corrected rGSH in the EBC. Similarly, concentrations of TA rGSH not normalized to urea moderately correlated with EBC rGSH concentrations not normalized to urea (*r* = 0.574, p = 0.002, 95% CI: (0.230-0.782), R^2^ = 0.329). Interestingly, the duration of mechanical ventilation demonstrated a moderate negative correlation with urea-corrected rGSH in the EBC (*r* = - 0.574, p = 0.003, 95% CI: (-0.779 – (-0.209), R^2^ = 0.329) but did not correlate with urea-corrected rGSH in the TA (p = NS). Figure 3 demonstrates a strong positive correlation between urea-corrected GSSG in the TA and urea-corrected GSSG in the EBC. A similarly strong positive correlation existed between TA GSSG concentrations not normalized by urea and EBC GSSG concentrations not normalized to urea (r = 0.708, p < 0.001, 95% CI: (0.431 – 0.856), R^2^ = 0.501). Although the fraction of oxidized GSH (i.e. the % GSSG) was approximately two times higher in the EBC than in the TA (EBC: 44.8 ± 4.6% vs TA: 21.8 ± 3%, p < 0.001), there was no significant correlation between the TA % GSSG and the EBC % GSSG (p = NS). When total GSH was compared between the two sampling sites, a moderate positive correlation was demonstrated in both urea-corrected values (Figure 4) and in non-urea normalized values (*r* = 0.649, p < 0.001, 95% CI: (0.338 – 0.824), R^2^ = 0.421).

### GSH status in both TA and EBC correlated with AM GSH staining

**Figure 5:**
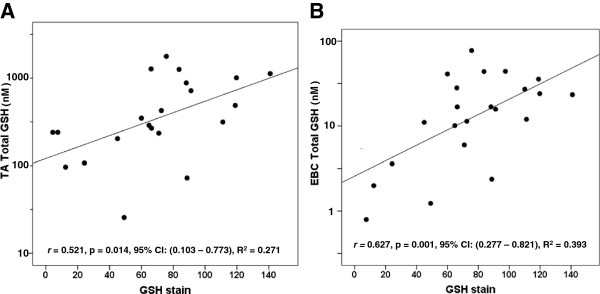
**Relationship between TA and EBC total GSH and AM GSH staining.** Total GSH concentrations in the **A)** TA and **B)** EBC as measured via HPLC were compared to GSH staining on the isolated AM as determined by immunofluorescence. **A)** A moderate positive correlation existed between TA total GSH and AM GSH stain. **B)** A moderately positive correlation also exists between EBC total GSH and AM GSH. Points represent the values of urea-corrected total GSH concentrations in nM on the Y axis versus AM GSH stain as quantified in relative fluorescent units (RFU)/cell on the X axis. Correlations were calculated after log_10_ normalization of the total GSH concentrations.

**Figure 6:**
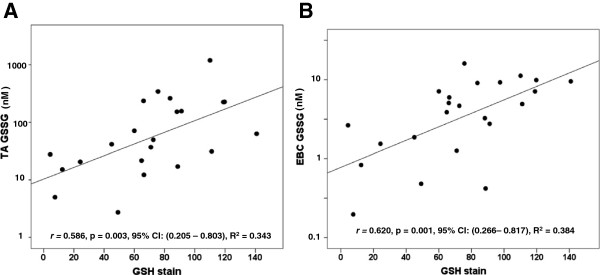
**Relationship between TA and EBC GSSG and AM GSH staining.** GSSG concentration in the **A)** TA and **B)** EBC were compared to GSH staining on the isolated AM. **A)** A moderate positive correlation existed between GSSG in the TA and AM GSH stain. **B)** A moderately positive correlation also exists between GSSG in the EBC and AM GSH. Points represent the values of urea-corrected GSSG concentrations in nM on the Y axis versus AM GSH stain as quantified in relative fluorescent units (RFU)/cell on the X axis. Correlations were calculated after log_10_ normalization of the total GSH concentrations.

Since we hypothesized that the GSH in the TA or EBC sample may be reflective of cellular GSH in the isolated AM, we evaluated the correlation of total GSH measured in TA and EBC with AM GSH staining determined via immunofluorescence. Fluorescent staining for protein-GSH adducts in the AM demonstrated a moderate positive correlation with the urea-corrected concentration of total GSH in the TA and the EBC (Figure 5A and B). Furthermore, AM demonstrated a moderate positive correlation with urea-corrected concentrations of GSSG in the TA (Figure 6A) and in the EBC (Figure 6B). While we did not observe a correlation between AM GSH and urea-corrected concentrations of rGSH in the TA (p = NS), a moderate positive correlation was found with urea-corrected concentrations of rGSH in the EBC (*r* = 0.571, p = 0.004, 95% CI: (0.195 – 0.791), R^2^ = 0.326).

## Discussion

In the current investigation, our main goals were 1) to determine if the status of the antioxidant GSH measured in a TA sample correlated with that of a corresponding EBC sample and 2) to determine whether EBC collection enabled a safe, non-invasive approach to evaluate such data in an intubated neonatal population. No adverse events where noted during EBC collection in our series of ventilated neonatal patients. Both the raw and urea-corrected concentrations of EBC rGSH, GSSG and total GSH significantly correlated with those measurements in the more invasive TA samples. Furthermore, this study demonstrated that both the total GSH and the GSSG in TA and EBC samples positively correlated with the cellular GSH staining of isolated AM.

Collection of EBC is based on the premise that airway surface liquid droplets are entrained and mixed with water vapor and other volatile gases during tidal breathing [17]. Upon exhalation, the breath is cooled in a passive sampling device which causes water vapor to condense into respiratory droplets plus other exhaled particles and collect on the chilled walls of the sampling device. Investigations into the use of EBC as a non-invasive tool to monitor pulmonary biomarkers of disease have gained much interest in the last decade [13]. Analysis of EBC has led to novel observations about the antioxidant status of the lung in varying disease states in both the adult and pediatric population [16,26]. Our laboratory has successfully used this method in previous studies to demonstrate diminished GSH availability in the lungs of asthmatic children [18] and adults [19].

Although EBC use has widely been studied with the hopes of utilizing this technique in clinical practice in the adult arena [12], data investigating EBC in newborn infants remains limited. Evaluation of EBC has been described in spontaneously breathing infants with a median age of approximately 2 years [27] and in older children between 1–30 months of age [28]. Collection of EBC via the exhalation limb of the ventilator has been reported in intubated pediatric patients [29] and in neonates requiring mechanical ventilation or CPAP [30]. However, the current study is the first report to our knowledge aimed at comparing TA and EBC samples in an exclusively intubated neonatal population. Our data suggest significant correlations existed between EBC and TA GSH markers in neonates, unlike studies in adult subjects, where EBC and BAL biomarkers such exhaled nitric oxide, 8-isoprostane and hydrogen peroxide did not correlate [31].

The strengths of the current study include a standardized approach to the collection of the EBC in terms of methodology and timing. EBC was collected from the exhalation limb of the ventilator in intubated newborns which reduced possible contamination from volatiles in the atmosphere or the mouth as has been demonstrated in spontaneously breathing subjects [12-14].

This study has several limitations. The goal of this project was to determine correlations between non-invasive EBC collection and TA collection; for this reason EBC and TA samples were drawn sequentially from each patient as to compare them to each other and not between population subsets. As such, the sample size of this pilot study was small. A much larger sample size would be required to appropriately control for gestational age, chronological age of the newborn, and respiratory diagnosis. However, data from this pilot study justifies a much larger, adequately powered clinical study aimed to define normative values in such a neonatal population.

During the study we did not monitor temperature stability throughout sample collections. The R-Tubes were precooled to -80°C for a minimum of 2 hours, kept in an insulated glove and placed on wet ice prior to and immediately after sample collection. However, the R tube has been shown to be sensitive to ambient temperatures and varying temperature has been noted to affect EBC analyses [13,14,32-34].

The standardization of EBC analyses remains limited since normalization for dilution within the EBC samples remains problematic. Current literature outlines an ongoing debate regarding the interpretation of dilution in EBC sampling [17,35-37]. While we attempted to control for dilution using the urea-correction, as is acceptable for BAL analyses, it remains unknown whether this method is applicable to EBC sampling [14]. Although the use of urea-correction for EBC is not yet established, our data suggest that this may be an appropriate method to address the effects of dilution in both TA and EBC sampling, since similar correlations were noted.

Investigations into the GSH status of premature newborns remain clinically important. Advances in modern neonatal respiratory support and clinical interventions for the prevention or treatment of BPD have not succeeded in reducing its occurrences [2]. Therefore, continued investigations into the antioxidant status of the at-risk premature newborn lung may allow patient-specific therapies to improve respiratory outcomes in this at-risk population [38]. Even though the thiol pair of GSH/GSSG is essential for normal cellular functioning and signaling in the lung [39], there remains limited information regarding its abundance in the premature newborn [8], who is particularly at risk for both inflammatory [40] and oxidative injury [6]. TA sampling, albeit less invasive than a formal BAL, still requires invasive suctioning of the airway. EBC holds potential as a non-invasive approach to evaluating GSH status in the neonatal lung, and to assist in identifying those particular infants at greatest risk for the development of BPD. The current study demonstrates feasibility of this approach in an intubated neonatal population. Interestingly, a moderate negative correlation was demonstrated between EBC rGSH and the duration of mechanical ventilation at the time of sample collection. Furthermore, since GSH availability modulates functioning of the resident AM [9,25], determining the status of GSH availability in the developing lung could advance our understanding of the mechanisms underlying antioxidant depletion and the development of BPD in the neonate.

## Conclusion

EBC collection in intubated neonates was well tolerated without significant changes in respiratory status. GSH status in the EBC positively correlated with that obtained in the more invasive TA, and positively correlated with that seen in isolated AM. Evaluation of EBC holds promise to monitor antioxidant status, such as GSH in premature newborns. Further investigations are required to incorporate this technology to assist in identifying those infants at greatest risk for subsequent oxidant-induced injury in the lung.

## Abbreviations

EBC: Exhaled breath condensates; TA: Tracheal aspirates; rGSH: Reduced glutathione, GSSG, glutathione disulfide; AM: Alveolar macrophage; NICU: Newborn intensive care unit; HPLC: High performance liquid chromatography; BPD: Bronchopulmonary dysplasia; BAL: Bronchoalveolar fluid; ECMO: Extracorporeal membrane oxygenation; IAA: Iodoacetic acid; BUN: Blood urea nitrogen level; IQR: Interquartile range.

## Competing interests

The authors have no financial ties or conflicts of interest to disclose.

## Authors’ contributions

Study idea and design: MR, SR, ET, LAB, TG; Patient enrollment and sample collection: MR, SR, ET, KR, TG; Sample processing and analyses: MR, XP, JW, KR; Statistical analyses and interpretation of data: MR, CM, LAB, TG. Manuscript drafting and revision: MR, CM, LAB, TG. All authors read and approved the final manuscript.
